# A comparison of bupivacaine lavage and diclofenac suppository effects on post-operative pain of laparascopic transabdominal pre-peritoneal herniorrhaphy: a randomized clinical trial study

**DOI:** 10.1186/s13104-020-05297-7

**Published:** 2020-10-01

**Authors:** Alireza Negahi, Seyed Hamzeh Mousavi, Vahid Abbasnezhad, Fatemeh Jahanshahi

**Affiliations:** 1 General Surgery Department, Rasoul Akram Hospital, Iran University of Medical Sciences, Tehran, Iran; 2 Student Research Committee, Faculty of Medicine, Iran University of Medical Sciences, Tehran, Iran

**Keywords:** Bupivacaine lavage, Diclofenac suppository, Postoperative pain, laparascopic, Herniorrhaphy, İnguinal hernia

## Abstract

**Objectives:**

Injection of a topical anaesthetic has been proved to be helpful with reducing pain after laparoscopic herniorrhaphy. We aimed to assess the effect of bupivacaine lavage on postoperative pain and compare it with diclofenac suppository. In this randomized clinical trial, 60 patients—scheduled for laparoscopic herniorrhaphy—were enrolled and randomized into three groups of 20 each, including diclofenac suppository, bupivacaine lavage, and normal saline as a placebo.The patients were investigated for postoperative pain scores, vomiting, nausea, morphine request, and duration of hospitalization.

**Results:**

In the bupivacaine group, pain levels in recovery room, 4, 8 and 12 h after surgery, were significantly lower than diclofenac group; at time points of 16, 20 and 24 h after surgery, difference between two groups was not significant. Regarding vomiting and nausea, at time points of 1 and 3 h after surgery, results show no significant difference between the groups. Incident of infection, 1 h and 1 week after the surgery, was not significantly different among the groups. Duration of hospitalization in the bupivacaine group was much lower than the diclofenac group. Based on our results, use of the bupivacaine lavage can reduce postoperative pain in patients undergoing laparoscopic herniorrhaphy.

*Trial Registration* Randomized clinical trial IRCT20180522039782N2; date of registration:14/10/2018

## Introduction

In recent years, repair of inguinal hernia with less invasive modern surgical techniques has minimized the possibility of relapse, and pared its acute postoperative pain, and improved patients’ satisfaction as well. However, despite the consistent advances, controlling postoperative chronic pain in this kind of surgery is still a challenge.

Additional beneficial effects of less invasive surgical methods, such as laparoscopy, include a reduction in hospitalization costs, enhancing patients’ quality of life, raising the standard of health care and faster re-engagement of the patients in daily activities [1]. However, the research to date has failed to resolve the issue of controlling postoperative pain and still no precise management algorithm has been introduced for it. This indicates the need for further attention to developing a multifaceted management approach for ameliorating postoperative pain of inguinal herniorrhaphy [2].

Non-steroidal anti-inflammatory drugs (NSAIDs) are a popular category of drugs for controlling postoperative pain which in short-term have the least side effects. In this category, analgesic diclofenac, reduces patient’s need to opioids after surgery and alleviates peritoneal pain and inflammation as well [3].

Another method for controlling postoperative pain is interapritoneal lavage with bupivacaine from surgical site which is sometimes exploited by surgeons [4, 5]. Bupivacaine is a long-acting local anesthetic drug which is very efficient in relieving postoperative pains. Although numerous studies have investigated several possible methods and drugs for controlling and relieving postoperative pain of laparoscopic transabdominal pre-peritoneal (TAPP) surgery of inguinal hernia, to the best of our knowledge, there is no record of a study in which the effects of bupivacaine and analgesic diclofenac have been compared [6–11]. In this randomized clinical trial, these two drugs are compared regarding their efficiency for pain after laparoscopic herniorrhaphy.

Additionally, a comparison has been drawn regarding duration of hospitalization, surgical wound infection, nausea, vomiting and request for rescue painkiller after the surgery.

## Main text

### Methods

For this study, a total of 60 patients were recruited from the General Surgery Department of Rasoul Akram Hospital during the year of 2018. According to the inclusion criteria of this study, male or female patients of American Society of Anesthesiologists Class 1 or 2 who were candidate for laparoscopic herniorrhaphy were included in the study. Also, due to anticipated medication side effects, pregnancy was considered as the exclusion criterion. Moreover, patients whose procedure was converted to open herniorrhaphy were excluded from the study. Prior to commencing the study, the patients were explained about the procedure and a written informed consent was obtained from them. All the included patient had underwent general anesthesia and had same anesthetic management without any administration of additional narcotic drugs, intraoperatively. The procedures of this study were approved by Ethical Committee of Iran Registry of University of Medical Science (IR.IUMS.FMD.REC1396.9311245006) and registered with Iran Randomized Clinical Trial Center (no: IRCT20180522039782N2).The study adheres to CONSORT guidelines for reporting this trial.

For the purpose of this study, according to the study of Suvikapakornkul et al. [12], the required sample size of each group was calculated as 20.

$${\text{n}} = \frac{{\left( {S_{1 }^{1} + S_{2}^{2} } \right)\left( {{\text{Z}}_{{1 - \frac{{\upalpha }}{2}}} + {\text{Z}}_{{1 - {\upbeta }}} } \right)^{2} }}{{\left( {\overline{X}_{1} - \overline{X}_{2} } \right)^{2} }}$$

$${\stackrel{-}{X}}_{1}\left(mean\, postoprative\, pain\, in \, one\, group\right)=3.5$$

$${\stackrel{-}{X}}_{2}\left(mean\, posoprative\, pain\, in\, the\, other\, group\right)=5.2$$

$${\mathrm{Z}}_{1-\frac{\mathrm{\alpha }}{2}}=1.96$$

$${\mathrm{Z}}_{1-\upbeta }=0.84$$

$${S}_{1}=1.5$$

$${S}_{2}=2.5$$

Study power = 90%

Patients in these groups were treated by bupivacaine, analgesic diclofenac and normal saline, separately. In the bupivacaine lavage group, the lavage was performed using a suction irrigation setting just before desufflation of the abdomen and closing the surgical site. In this group, 0.25% bupivacaine in 100 ml of physiological serum was used.

The patients were placed in the supine position and bupivacaine lavage was performed through the umbilical into the abdominal cavity. A diclofenac suppository 50 mg was prescribed for the diclofenac group every 8 h; the first dose was administrated immediately after the surgery. One g IV stat of paracetamol was infused for all the patients after the surgery. A questionnaire including variables such as postoperative visual analogue scale (VAS), vomiting, nausea, age, gender, request for rescue morphine, duration of hospitalization was completed by a staff member for each patient. These variables were registered at time points of 0 (in the recovery room), 4, 8, 12, 16, 20, and 24 h after the surgery. Then, the collected data were analyzed and interpreted using SPSS version 22. To analyze qualitative variables, frequency and percentage were reported and compared using chi-square test. Regarding quantitative variables, the mean and standard deviation were presented.

Comparisons between the quantitative variables were done using the *t*-test (for normal distribution), and the Mann-Whiteney U-Test (for abnormal distribution), was used. To compare the groups, the Analysis of Variance (ANOVA) or the Kruskal Wallis was used. A *P* value less than of 0.05 was considered significant.

### Results

**Fig. 1:**
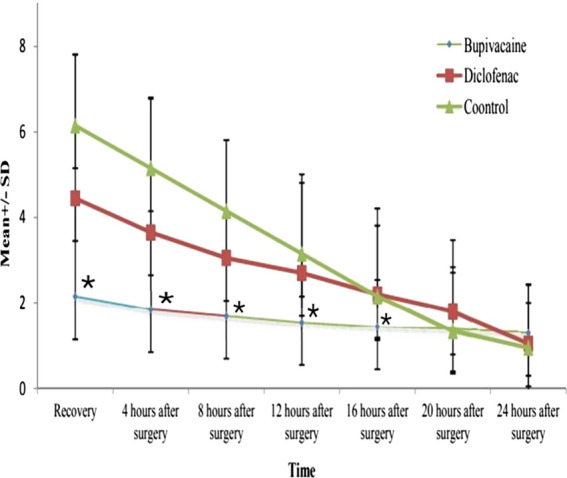
The trend of VAS score changes over the assessed times

In this randomized clinical trial, 60 patients were divided into three groups of 20 each. The first group, consisted of 16 men (80%) and 4 women (20%) with mean age of 47.05, were administered with bupivacaine lavage, and the second group, consisted of 17 men (85%) and 3 women (5%) with mean age of 51.75 received analgesic diclofenac. The control group consisted of 18 men (90%) and 2 women (10%) with mean age of 48.1 and they were treated with normal saline (Table1). The comparison of the analyzed data— collected at the time points of 0 h (at recovery room), 4, 8, 12, 16, 20, 24 h after the surgery— revealed a significant difference between the three groups regarding pain levels experienced (P < 0.05). Pair wise comparisons also indicated that in the bupivacaine group, postoperative pain levels were significantly less than the analgesic diclofenac group and the control group (P < 0.05). Moreover, the pain levels of the analgesic diclofenac group were less than those of the control group. However, none of the differences at the time points of 16, 20 and 24 h after the surgery, between the three groups, were statistically significant; accordingly, the pain levels of the bupivacaine group and analgesic diclofenac group were not significantly different at these time points. The mean postoperative pain at the time points of 0, 4, 8, 12, 16 h after the surgery was significantly different between the three groups. Pair comparison of the groups showed that the mean postoperative pain was significantly lower in the bupivacaine group compared with the diclofenac and control groups (*P* value ˂ 0.05) (Fig. 1).

**Table 1:** The comparison of age and gender between the 3 groups

Characteristics	Groups			*P* value
	Bupivacaine lavage	Diclofenac suppository	Control	
Age(year)				
SD ± Average	47.05 ± 13.1	51.75 ± 11.23	48.1 ± 11.25	0.429
Sex				
Male	16 (80%)	17 (85%)	18 (90%)	0.676
Female	4 (20%)	3 (15%)	2 (10%)	

There were no statistically significant differences between the age and gender of the patients (*p* value > 0.05) in the 3 groups

To draw a comparison between the three groups in terms of experiencing nausea and vomiting, the Chi-Square Test method was used. This comparison disclosed that at the time point of 1 h after the surgery, the three groups were not significantly different, regarding the incident of nausea and vomiting (*P* > 0.05). Similarly, at the time point of 3 h after the surgery, no difference was found between the groups (Table 2).

**Table 2:** The comparison of postoperative nausea and vomiting between the 3 groups

Time	Groups			Nausea and vomiting	*P* value
	Bupivacaine lavage	Diclofenac suppository	Control		
1 h postopration	3 (15%)	3 (15%)	4 (20%)	Yes	0.887
	17 (85%)	17 (85%)	16 (80%)	No	
3 h postopration	1 (5%)	1 (15%)	3 (15%)	Yes	0.418
	19 (95%)	19 (95%)	17 (85%)	No	

There were no statistically significant differences in nausea and vomiting 1 and 3 hr after surgery between the 3 groups

Regarding the incident of infection 1 week after the surgery, the Chi-Square Test showed that the three groups did not differ significantly (*P* > 0.05) (Additional file 1. Table S1).

The Chi-Square Test was applied to studying the request for rescue pain killer in the three groups. While the demand for rescue painkiller was higher in the control group (*P* < 0.05), the results confirmed that, undoubtedly, there was a positive association between the use of bupivacaine lavage and request for painkiller (*P* < 0.05) (Additional file 1. Table S2). The Kruskal Wallis test was used to determine and evaluate the hospitalization periods of the patients; the results, as shown in (Additional file 1. Table S3), interestingly demonstrate a significant difference between the groups (*P* < 0.05). Moreover, pair wise comparisons assert that the patients in the bupivacaine lavage group required a significantly less hospitalization period (*P* < 0.05when compared with the analgesic diclofenac group. Certainly, the hospitalization time in the bupivacainelavage group was less than that in the control group (*P* < 0.05).

### Discussion

The repair of TAPP and total extraperitoneal (TEP) repair are two of the most frequent laparoscopic repair methods for inguinal hernia. Laparoscopic repair of inguinal hernia should be performed by a surgeon familiar with traditional anterior repair and laparoscopic technique as well as an extensive insight into anatomy of the inguinal general area and posterior view of the anterior abdominal wall [1]. With improving the laparoscopic techniques and surgeon’s skills, the probability of relapse can be reduced to the extent reported for the traditional techniques of hernia repair. Postoperative pain is recognized as an undesirable outcome and widely discussed in the literature. However, none of them can be considered as an efficient method to reducing postoperative pain. Unfortunately, there is still a little understanding about the contributing factors, leading to various pain syndromes before, during and after the surgery. Actually, depending on the root cause, these syndromes could be intrinsically somatic or visceral and their treatment might be complicated [2].

The present study aimed to determine the effect of the bupivacaine lavage and diclofenac suppository on postoperative pain in laparascopic herniorrhaphy and to compare them which each other.

In our study, pain scores and hospitalization periods were considerable improved in the patients treated with bupivacaine lavage,, compared to the patients treated with diclofenac suppository.

Gupta et al. (2016) compared ropivacaine 0.25% and 0.5% with bupivacaine 0.25% in a group of 90 patients underwent inguinal herniorrhaphy and they observed that postoperative VAS pain scores for the bupivacaine 0.25% group improved. Their findings, consistent with our findings, confirmed that bupivacaine causes no nausea and vomiting as side effects [13]. Savestani et al. (2013) examined the pain relieving effect of intraperitoneal instillation of hydrocortisone and compared it with normal saline. The numerical rating scale (NRS) pain scores in hydrocortisone group were significantly lower than normal saline group. Moreover, the hydrocortisone group showed less demand doses of pain killer besides no sign of nausea and vomiting [14].

Surveys such Saeed Safari et al.’ study (2020) have shown that individuals, who received intraperitoneal lavage of bupivacaine 0.2% in laparascopic bariatric surgeries, experienced lower pain scores on coughing and resting compared with those in placebo group. Furthermore, the studies have shown that intraperitoneal lavage of bupivacaine is a simple and an effective technique for controlling the postoperative pain of laparascopic bariatric surgeries [15].

Moreover, a considerable amount of previous studies have reported the positive effects of bupivacaine on alleviating pain after laparascopic cholecystectomy. For instance, Abdul Manan et al. in 2020, in a randomized clinical trial, investigated 55 patients underwent laparoscopic cholecystectomy for the effect of bupivacaine against a 55-patient placebo group. Based on finding of this study, we conclude that analgesia duration after the surgery in bupivacaine group was significantly higher than placebo group [16]. Suma S. et al. in 2019 treated two groups of 34- patient separately with bupivacaine lavage and normal saline lavage as placebo at the gall bladder fossa.

Moreover, our finding led us to conclude that bupivacaine lavage was more effective on controlling postoperative pain of laparascopic cholecystectomy compared with the placebo. The mean of pain score in the bupivacaine group was significantly less than the placebo group [17]. The consistent results of these studies and their findings support the idea of potency of lavage method in relieving postoperative pain of laparascopic surgeries; nevertheless, there are a few limitations and the results are not always as desired. Further investigations are required to explore safer and more effectual drugs for alleviating postoperative pains.

## Limitations

This study extends our knowledge on postoperative pain relieving methods; however, the findings were limited because of the use of a relatively small size group; therefore, they should be interpreted with caution. In this regard, it is recommended to perform further researches with larger group sizes.

## Supplementary information

**Additional file 1:** **Table S1.** The comparison of postoperative infection between the three groups. **Table S2.** The comparison of request of rescue pain reliever between the three groups. **Table S3.** The comparison of hospitalization duration between the three groups.

## Data Availability

The datasets used and analysed during the current study are available from the corresponding author on reasonable request.

## Abbreviations

NSAIDs: Non- steroidal anti-inflammatory drugs
VAS: Visual analogue scale
TAPP: Transabdominal pre-peritoneal
TEP: Total extra-peritoneal
NRS: Numerical rating scale
ANOVA: Analysis of Variance Test

## References

[1] Donmez T, Erdem VM, Sunamak O, Erdem DA, Avaroglu HI (2016). Laparoscopic total extraperitoneal repair under spinal anesthesia versus general anesthesia: a randomized prospective study. Ther Clin Risk Manag.

[2] Gould J (2008). Laparoscopic versus open inguinal hernia repair. Surgl Clin North.

[3] Yarani M, Amouzeshi A, Behmanesh M, Pourbagher-Shahri AM, Hozeifi S, Rajabpour-Sanati A (2019). Comparison of the effect of diclofenac suppository with intravenous meperidine in relieving pain after laparoscopic cholecystectomy in opioid-dependent and independent patients. J Surg Trauma.

[4] Najmaldin A, Guillou PJ (1998). A guide to laparoscopic surgery.

[5] Nathaniel J, Leel S, Swanstron W, Eubanks S, Robert J, Fitzgibbons J, Lee R (2005). Laparoscopictransabdominalpreperitoneal repair of inguinofemoral hernias. Mastery of endoscopic andlaparoscopic surgery.

[6] O’Riordain DS, Kelly P, Horgan PG, Keane FB, Tanner WA (1998). A randomized controlled trial of extraperitoneal bupivacaine analgesia in laparoscopic hernia repair. Am J Surg.

[7] Alkhamesi NA, Peck DH, Lomax D, Darzi AW (2007). Intraperitonealaerosolization of bupivacaine reduces postoperative pain in laparoscopic surgery: a randomized prospective controlled double- blinded clinical trial. Surg Endosc.

[8] Sripada S, Roy S, Mathur M, Hamilton M, Cranfield K, Bhattacharya SA (2006). prospective double-blind randomized controlled trial of intraoperative pelvic instillation withbupivacaine for management of pain following laparoscopyand dye. Br J Obstetet Gynaecol.

[9] Saff GN, Marks RA, Kuroda M, Rozan JP, Hertz R (1998). Analgesic effect of bupivacaine on extraperitoneal laparoscopic hernia repair. Anesth Analg.

[10] Deans GT, Wilson MS, Brough WA (1998). Controlled trial ofpreperitoneal local anesthetic for reducing pain followinglaparoscopic hernia repair. Br J Surg.

[11] Bar-Dayan A, Natour M, Bar-Zakai B, Zmora O, Shabtai M, Ayalon A, Kuriansky J (2004). Preperitonealbupivacaine attenuates pain following laparoscopic inguinalhernia repair. Surg Endosc.

[12] Suvikapakornkul R, Valaivarangkul P, Noiwan P, Phansukphon T (2009). A randomized controlled trial of preperitoneal bupivacaine instillation for reducing pain following laparoscopic inguinal herniorrhaphy. Surg Innov.

[13] Gupta SL, Bidkar PU, Adinarayanan S, Prakash MS, Aswini L (2016). Postoperative analgesia after inguinal hernia repair-Comparison of ropivacaine with bupivacaine: a randomized controlled trial. Anesthia Essays Res.

[14] Sarvestani AS, Amini S, Kalhor M, Roshanravan R, Mohammadi M, Lebaschi AH (2013). Intraperitoneal hydrocortisone for pain relief after laparoscopic cholecystectomy. Saudi J Anaesth.

[15] Safari S, Rokhtabnak F, Motlagh SD, Garkani MG, Pournajafian A (2020). Effect of intraperitoneal bupivacaine on postoperative pain in laparoscopic bariatric surgeries. Surg Obes Related Dis.

[16] Manan A, Khan AA, Ahmad I, Usman M, Jamil T, Sajid MA (2020). Intraperitoneal bupivacaine as post-laparoscopic cholecystectomy analgesia. J Coll Physcians Surgeons Pakistan.

[17] Suma S, Vikranth Suresh N, Nikhil M, Sreeramulu PN (2019). Intra-peritoneal bupivacaine instillation for post-operative pain relief after laparoscopic cholecystectomy: a prospective study. Int J Contemp Surg.

