# Supplementary material for: A comparison of bupivacaine lavage and diclofenac suppository effects on post-operative pain of laparascopic transabdominal pre-peritoneal herniorrhaphy: a randomized clinical trial study
Source: BMC Res Notes. 2020 Oct 1;13:463. doi: 10.1186/s13104-020-05297-7 (PMC7528389; doi:10.1186/s13104-020-05297-7)
Supplement: Supplementary file 1 — Additional file 1: Table S1. The comparison of postoperative infection between the three groups. Table S2. The comparison of request of rescue pain reliever between the three groups. Table S3. The comparison of hospitalization duration between the three groups. [file 13104_2020_5297_MOESM1_ESM.docx]

Table S1: The comparison of postoperative infection between the three groups

| Time | Groups | | | Infection | P_Value |
| --- | --- | --- | --- | --- | --- |
|  | Bupivacaine  Lavage | Diclofenac  Suppository | Control |  |  |
| 1 week  Postopration | 0(0%)  20(100%) | 0(0%)  20(100%) | 0(0%)  20(100%) | Yes  No | 1 |

There were no statistically significant differences in terms of infection incidence 1hr and 1week after surgery between the three groups.(p value>0.05).

Table S2: The comparison of request of rescue painkiller between the three groups

| Characteristic | Groups | | | P_Value |
| --- | --- | --- | --- | --- |
|  | Bupivacaine  Lavage | Diclofenac  Suppository | Control |  |
| Pain reliever  Need | 0(0%)  20(100%) | 2(10%)  18(90%) | 18(90%)  2(10%) | <0.001 |

The need for analgesics was significantly higher in the control group than the other two groups (p value<0.05).

Table S3: The comparison of hospitalization duration between the three groups

| Characteristic | Groups | | | P_Value |
| --- | --- | --- | --- | --- |
|  | Bupivacaine  Lavage | Diclofenac  Suppository | Control |  |
| Hospitalization  Duration(day)  SD±Mean | 1.15±0.36 | 2.4±1.72 | 3.3±1.55 | <0.001 |

Hospitalization duration in the bupivacaine group was significantly lower than the other two groups.
